# Spatial-Temporal Distribution of 12-Year Periodontal Disease Prevalence in a Large Population Using Geographical Information Systems: A Longitudinal Study

**DOI:** 10.3290/j.ohpd.b5816556

**Published:** 2024-11-07

**Authors:** Bulent Bostanci, Gozde Erimli, Duygu Kilic

**Affiliations:** a Associate Professor, Department of Geomatics Engineering, Faculty of Engineering, Erciyes University, Kayseri, Turkey. Study concept, formal analyses, investigation, methodology, project administration, wrote, reviewed and edited manuscript, validation, visualisation, software.; b Periodontist, Department of Periodontology, Faculty of Dentistry, Erciyes University, Kayseri, Turkey. Study concept, formal analyses, investigation, methodology, project administration, wrote, reviewed and edited manuscript, validation, visualisation.; c Assistant Professor, Department of Periodontology, Faculty of Dentistry, Erciyes University, Kayseri, Turkey. Study concept, formal analyses, investigation, methodology, project administration, wrote, reviewed and edited manuscript, validation.

**Keywords:** epidemiology, geographic mapping, gingivitis, periodontitis, spatial analysis.

## Abstract

**Purpose:**

Periodontal diseases, commonly linked to dental biofilm and affecting adults, were studied using Geographic Information Systems (GIS) and Kernel Analyses with epidemiological data. This paper presents a hybrid method for use in epidemiological studies by evaluating the spatiotemporal distribution of disease prevalence.

**Materials and Methods:**

This study analy ed 47,757 patients from the Department of Periodontology out of 662,351 visitors to University Faculty of Dentistry (2012 to July 2023). The central districts of Kayseri in Turkey were selected as the study areas. Periodontitis prevalence was assessed through radiographic evidence and clinical examination. Point-based location data, including gender, age, and disease type, matched household data, creating building-based spatial data. Kernel Density (KD) and Average Nearest Neighbor (ANN) analyses examined patient concentration and disease types in specific regions. Accordingly, standard deviation ellipses were prepared by year to assess the spatial changes in the regions where patients resided.

**Results:**

The study found higher periodontitis prevalence in males, increasing with age, while gingivitis decreased. After 2017, periodontitis prevalence notably declined. Location-based data exhibited clustering in patient distribution. KD maps showed similar patient distributions over the years, with more applications from areas closer to the Faculty of Dentistry. The spatial distribution of the patients applying has remained consistent over the last 5 years.

**Conclusions:**

Through GIS, KD maps reveal the spatial-temporal distribution of periodontitis patients. This aids in identifying high-prevalence regions and guiding strategic healthcare facility placement. Implementing preventive programs in high-demand areas, particularly in family health centers (local health facilities), can reduce community-wide periodontal disease prevalence.

Periodontal diseases are a significant public health problem primarily affecting adults and are highly prevalent, affecting approximately 50%–90% of the world’s population. They are often associated with dental biofilm.^11,15^ Epidemiological studies in periodontology are crucial for identifying individuals at risk of disease and developing preventive and protective treatment programs accordingly.^22^ Understanding how the disease is defined, its prevalence, severity, and treatment, coupled with population-based data, allows for improving overall periodontal health in the community. It facilitates the development of preventive health programs and ensures a more effective allocation of government resources for health policies.^11^

Gingivitis can be defined as a limited inflammation of the gums and is more common than periodontitis. It is a reversible condition when treated. Periodontitis is a chronic disease that destroys the supporting alveolar bone, periodontal ligament, and cementum tissues, accompanied by an inflammatory immune response. In contrast to gingivitis, periodontitis is characterised by alveolar bone loss on radiographs.^21,29^

The Geographic Information System (GIS) is a technological system used to collect, store, process, analyse, and visualise geographical data. Its fundamental components include geographic data, database management, map production, analysis, processing, and visualization. GIS is a powerful tool that aids in better understanding and effectively utilising data within a geographic context.^39^ Geographic data is widely used for management, analysis, and making more effective decisions in various fields such as urban planning, transportation, healthcare, and education. There are some important studies conducted in the healthcare field using GIS in Turkey.^7-9,27,32^

Kernel Density (KD) can be defined as a non-parametric estimation of continuously deriving a surface using various density functions.^36^ KD enables the use of point, line, and area data in GIS-based studies. Therefore, KD is beneficial for empirical studies where observations are represented by two-dimensional points containing the home addresses of individual patients.^20^

Spatial density analyses have also been used in some studies in the field of dental health. Jeong et al^19^ examined patient distribution in terms of proximity, accessibility, age, gender, and socioeconomic status using GIS and regression analyses. Eke et al^10^ utilised NHANES data in the USA to predict periodontitis prevalence at both the local and state levels using multilevel regression analyses. De Paiva et al^6^ assessed the spatial density and independent interest variables by residence of 14-year-old adolescents who experienced dental trauma in the city for Diamantina, Brazil, using Ripley K function and KD analysis. Nayak et al^26^ examined the applications of GIS-based spatial analyses in dentistry in their study titled “Geographic Information Systems in Spatial Epidemiology: Unveiling New Horizons in Dental Public Health.” However, they did not conduct a practical, application-based study.^26^ Literature reviews yield very few studies in dentistry that utilise GIS-based spatial analyses and KD analyses to investigate the spatial-temporal distributions of diseases for epidemiological purposes. Additionally, the absence of any other study assessing the prevalence of periodontal disease using these analyses makes our research unique.

This cross-sectional study uses spatial analyses to assess the 12-year prevalence of gingivitis and periodontitis in a broad population. By creating population density distribution maps, the study aims to guide the construction and placement of local health facilities, enabling regional access to preventive health programs based on the identified needs. The ultimate goal is to contribute to shaping future health policies.

## MATERIALS AND METHODS

**Fig 1:**
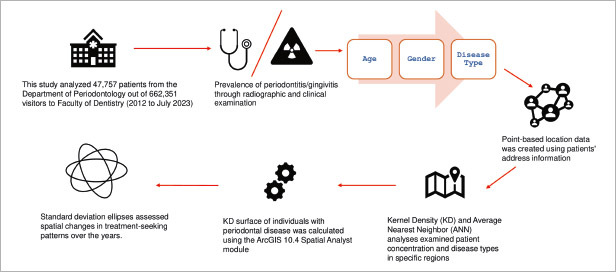
Study workflow.

This study used the clinical records of patients diagnosed with periodontal disease who applied to the Oral Diagnosis and Radiology Clinic of the Faculty of Dentistry in Kayseri, Turkey, between July 2012 and 2023. In Turkey, patients can apply to dental faculties to receive treatment when they have any dental complaints. Patients diagnosed by specialists at the Oral Diagnosis and Radiology Clinic are referred to other departments within the Faculty of Dentistry based on their treatment needs. Between 2012 and 2023, 662,351 patients visited Faculty of Dentistry, and 61,604 patients sought treatment at the periodontology clinic. Those with missing address information, applicants from other provinces, and patients from districts outside of the central districts totaling 13,847 were excluded from the study. After exclusion based on the criteria, a total of 47,757 patients were finally analysed. The prevalence of periodontitis was determined through radiographic evidence of bone loss recorded in panoramic radiographs (OPG: orthopantomograms) and bitewing radiographs (BW), along with clinical examination records. In a healthy individual, the crest of the alveolar ridge positions horizontally 1–2 mm apical to an imaginary line drawn at the cementoenamel junction. Patients applied to the Oral Diagnosis and Radiology Clinic of Faculty of Dentistry are diagnosed with periodontitis based on the following criteria: more than 2 mm of bone loss in radiography (≥2 teeth) and more than ≥1–2 mm of clinical attachment loss (CAL) during clinical examination in the interproximal area of any ≥2 non-adjacent teeth.^13,33^ Based on this, patients were categorised into periodontitis and gingivitis. The examinations and diagnoses were conducted by multiple specialists training in the Oral Diagnosis and Radiology Clinic of the Faculty of Dentistry. A Williams-Hu Friedy probe was used to assess the periodontal condition during clinical examinations. Patients outside the age range of 18 to 65 were excluded from the study. Patients with indications for “periodontal consultation” and other diagnoses such as “periodontal abscess,” “gingival abscess,” and “gingival recession” were categorised as “other” rather than classified as gingivitis/periodontitis. The records regarding patients’ addresses and personal information were obtained from the Dentistry Faculty’s information technology unit. The patients’ identity and address information were kept confidential. This study was approved by University Faculty of Medicine Medical Research Ethics Committee with the decision numbered 2023/784 on 06.12.2023. The study adhered to the tenets of the Declaration of Helsinki. The manuscript adheres to the STROBE reporting guidelines. Our study is a cross-sectional, retrospective investigation focused on diagnosis, and no interventional procedure was performed on the patients; therefore, informed consent forms were not obtained. Only records of past clinical examinations, radiographic images, and address information of patients who had applied to the Faculty of Dentistry over the years were used. The workflow of the study is shown in Fig 1.

## Statistical Analysis

### Spatial and temporal distribution analyses

In our study, the following methods were employed to conduct spatial and temporal distribution analyses.

#### Average Nearest Network (ANN)

**Analysis of settlement distribution models using ANN.** The ANN is an ArcGIS tool used to calculate the average distance-based nearest neighbour index between each feature and its nearest neighbour based on each feature’s average distance to its nearest neighbours.

The ANN (Spatial Statistics) Tool calculates the nearest neighbour index based on the average distance from each feature to its nearest neighbour. This is done for all crime incidents and then separately for individual types of crimes. The ANN tool returns five values: Observed Average Distance, Expected Average Distance, Nearest Neighbor Ratio, z-score with standard deviation, and probability with a p-value. The z-score and p-value values indicate whether the features exhibit a random pattern or statistically significant clustering or distribution.^4^

#### Kernel Density

The KD estimate is a non-parametric statistical interpolation method transforming point events into density surfaces.^16^ The kernel function is fitted to the observed data points, and the local density around the data points is examined.^2^ Using the Kernel Function, a value is calculated per unit area for point or multiple line features to fit a smooth cone-shaped surface to each point or multiple lines. Barrier lines of a specific type can be used to modify the influence of a feature when calculating kernel density. The general form of the KD function is as follows:


$$\hat{\lambda }(S)=\overset{n}{\underset{i=1}{\sum }}\frac{1}{\tau^{2}}\cdot k\cdot \frac{\left(s-s_{i}\right)}{\tau }$$

where the value λ̂ (*S*) represents the estimated density of the spatial point pattern at location s; si is the observed first event, k represents the kernel weighting function, and τ is the bandwidth.

In GIS-based analyses, Kernel Density calculates the density of point features around each output raster cell. Conceptually, a smoothly curved surface is placed over each point. The surface value is highest at the location of the point and decreases as you move away from the point, reaching zero at the search radius distance from the point. Only a circular neighborhood is possible. The volume below the surface equals the population area value for the point, or 1 if „NONE“ is specified. The density in each output raster cell is calculated by summing the values of all kernel surfaces at the locations where they cover the center of the raster cell. The kernel function is based on the quartic kernel function described by Silverman. If a population area setting other than “NONE” is used, the value for each item determines how many times the point should be counted. For example, a point with a value of 3 would be counted as three points. The values can be integers or floating-point numbers.^3^

In GIS-based analyses, the estimated density at a new (x, y) location is determined by the following formula:


Density=1( radius )2∑i=1n[3π⋅ pop i⋅(1−( dist i radius )2)2]

where:

- i = input points for 1, 2, .., n. Only points within the radius distance of the (x, y) location are included in the summation.
- pop_i_ is the optional parameter representing the population area value for the specific point i.
- disti is the distance between point i and the (x, y) location.

The calculated density is then multiplied by the count of points or the sum of the Population Field if entered. This correction ensures that the spatial integral equals the count of points (or Population Field) rather than always being equal to 1. This implementation uses a quartic kernel.^30^ The formula should be calculated for each location where you want to estimate the density. When creating a raster, the calculations are applied to the center of each cell in the output raster.^18^

When running the KD tool, it creates a single smooth density surface layer over the input points. The output density will vary based on the parameters entered in the tool, including the search radius and cell size used.

#### Considerations for determining the default search radius (bandwidth)

The average center of input points is calculated. If a Population Field value is entered, all subsequent calculations will be weighted based on the values in this field.

- The (weighted) average distance to the mean center is calculated for all points.
- The (weighted) median of these distances (Dm) is calculated.
- (Weighted) Standard Distance (SD) is calculated.

There are numerous studies regarding the selection of the bandwidth for KD. While some researchers argue that the bandwidth should not exceed the desired optimal resolution, others prefer to base the KD bandwidth on nearest neighbour distances.^23^ Some researchers have discussed the specific kernel bandwidth size to produce a smoother or coarser kernel.^14,31^ However, there is no complete consensus in the literature on how the bandwidth size should be selected.^23^

Some GIS-based programs suggest the following formula for calculating the bandwidth:^18^


$$\text{ SearchRadius }=0,9*\min\left(SD,\sqrt{\frac{1}{\ln(2)}}*Dm\right)*n^{-0.2}$$

In this formula:

- „min“ means that only the smallest value of the two resulting options will be considered.^18^
- Dm is the (weighted) median distance from the (weighted) mean center.
- n is the sum of the population area values (the count of points when no population area is used or the sum of the population area when provided).
- SD is the standard distance.

#### Analysis of the Temporal and Spatial Trends of Periodontal Diseases

Ellipses with measures of central tendency, dispersion, and directions, indicated by standard deviations, are created to determine geographic data’s temporal and spatial changes.

**Supplementary Fig 1:**
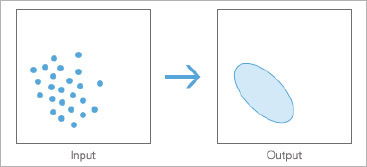
Suppose standard deviation ellipses are drawn based on the date feature of patient population data. In that case, the spatial distribution changes of the patient population can be assessed over time (daily, monthly, yearly).^17^

The obtained ellipse is used to assess whether the temporal distribution of the data changes in the X and Y directions and, therefore, whether it has a specific orientation angle (supplementary Fig 1).^17^

### Study area

**Supplementary Fig 2:**
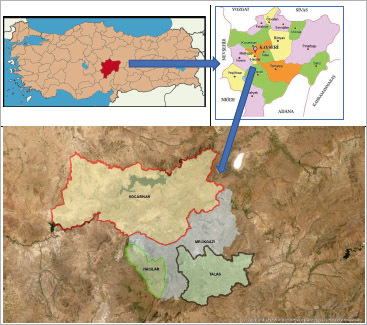
According to the 2022 address-based population registration system, the total population of Kayseri‘s four central districts is 1,185,000 people.

The central districts of Kayseri; Melikgazi, Kocasinan, Talas, and Hacılar were selected as the study areas (supplementary Fig 2).

## RESULTS

### Demographic Data

Between 2012 and 2023, 63.14% of the patients who were treated at the periodontology clinic were women, while 36.86% were men (Table 1).

**Table 1:** Distribution of periodontal diseases by age groups and gender

	All Patients	Gingivitis n (%)	Periodontitis n (%)	Other*
Gender				
Male	17.608 (36.86%)	8.302 (47.15%)	7.215 (40.97%)	2.091 (11.88%)
Female	30.149 (63.14%)	16.532 (54.83%)	10.172 (33.74%)	3.445 (11.42%)
Age range				
18–25	8522	7620 (89.41%)	427 (5.02%)	475 (5.57%)
26–35	10376	7258 (69.94%)	2332 (22.48%)	786 (7.58%)
36–45	13138	6016 (45.79%)	5613 (42.73%)	1509 (11.48%)
46–55	10140	2877 (28.37%)	5702 (56.24%)	1561 (15.39%)
56–65	5581	1063 (19.05%)	3577 (64.09%)	941 (16.86%)
Total	47757	24834 (52%)	17651 (36.96%)	5272 (11.04%)

*Complaint of pain, surgical consultation, periodontal consultation, gingival abscess, periodontal abscess, gingival growth, gingival recession, frenectomy.

Between 2012 and July 2023, among female patients who attended the periodontology clinic, 54.83% were diagnosed with gingivitis, and 33.74% were diagnosed with periodontitis. For male patients during the same period, 47.15% were diagnosed with gingivitis, and 40.97% were diagnosed with periodontitis (Table 1).

As the age range moves from 18-25 to 55-65, there is a decrease in the frequency of gingivitis and an increase in periodontitis, as found in Table 1.

**Fig 2:**
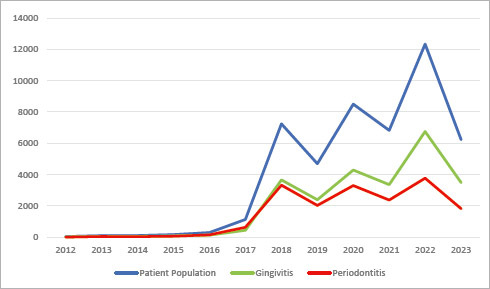
The distribution of patients visiting the periodontology clinic from 2012 to July 2023.

The distribution of patients visiting the periodontology clinic from 2012 to July 2023 is shown in Fig 2. The distribution of the gingivitis and periodontitis patient populations is graphically quite similar over the years.

**Fig 3:**
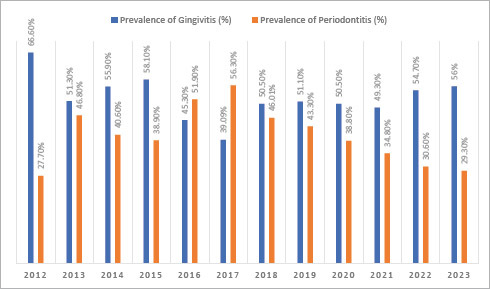
The distribution of gingivitis and periodontitis prevalence from July 2012 to July 2023.

From 2012 to July 2023, no statistically significant change was observed in the prevalence of gingivitis, but a notable decrease in periodontitis has been observed since 2017 (Fig 3).

### GIS Based Analyses

Kernel Density Estimation and the ANN method are used in many fields, including ecology, epidemiology, urban planning and geography, as they help understand and visualise the distribution of spatial data structure and determine whether points in the dataset are randomly distributed, clustered or uniformly distributed throughout a study area.

In the light of this information, density analyses may not have sufficient significance due to the low number of applications from districts outside the central districts and from other provinces. Therefore, we excluded the data of patients from these groups (61,604 patients) from the density analyses.

**Fig 4:**
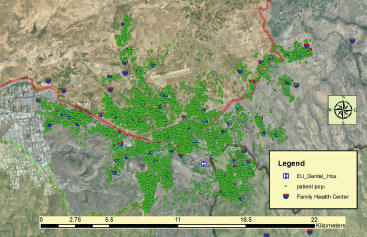
The data of 47,757 patients who applied to the Periodontology Clinic of Erciyes University Faculty of Dentistry between 2012 and July 2023 were obtained in Excel format from the patient registration system. Text data indicating addresses in the Excel table were matched with household information from the Kayseri Metropolitan Municipality Geographic Information Systems Directorate and transformed into point-based location data. In the second stage of the analysis, the KD surface of individuals with periodontal disease was calculated using the ArcGIS 10.4 Spatial Analyst module. It is essential to correctly determine the bandwidth and cell size to produce the most accurate density map in the KD method. The suggested cell size of 83x83 meters by the ArcGIS 10.4 program has been used as the standard value in producing Kernel Density maps. Choosing an appropriate bandwidth in KD estimation is a crucial step in the analysis because it significantly influences the resulting density prediction. The bandwidth will determine the smoothing degree (smoothness and level of detail) of the generated map.^14^ While some researchers argue that the bandwidth should not exceed the desired optimal resolution, others prefer to base the KD bandwidth on nearest neighbour distances.^23^ There is no complete consensus on the bandwidth issue. Experimenting with different bandwidths and evaluating their impact on the results will help obtain the correct density map. Therefore, Kernel Density maps were produced using bandwidth values of 500, 1000, and 1500 meters in the ArcGIS 10.4 program.

The point-based location data of patients and the locations of family health centers and the Faculty of Dentistry are shown in Fig 4.

**Supplementary Fig 3:**
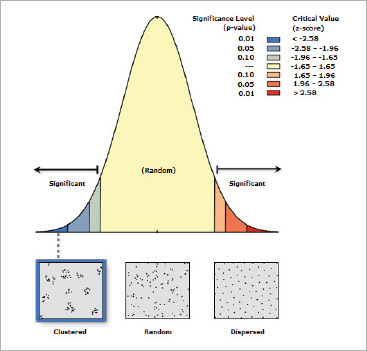
A distribution analysis of patient data has been created in the first analysis stage. Upon examination of the values, the observed mean distance is 6.49 meters, the expected mean distance is 54.61 meters, and the nearest neighbour ratio is 0.118905. The ratio being close to 0 indicates that the data distribution is clustered. The Z-score value being less than (-368.36)-2.58 and the p-value being 0.0000 invalidate the hypothesis that the data is random.

Before the KD analysis, a distribution analysis of the patient population data was created in the first analysis stage. The patient population data was evaluated using the ANN method and it was observed that the statistical distribution of patients exhibited a clustered structure (supplementary Fig 3).

In the second stage of the analysis, the KD surface of individuals with periodontal disease was calculated using the ArcGIS 10.4 Spatial Analyst module. The KD map prepared using GIS depicts areas with a high-density patient population in red and orange colors (Fig 5a). The comparative results are presented in Figs 5b and 5c. It is observed that the density maps obtained for Gingivitis and Periodontitis diseases are remarkably similar to each other.

**Fig 5a:**
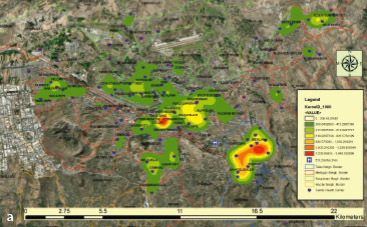
Among the created maps, Fig 4a presents the Kernel Density map with a bandwidth of 1000 meters, providing the best smoothness and neighbourhood-level detail.

**Fig 5b and 5c:**
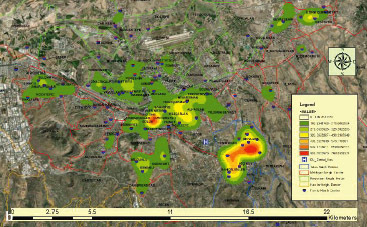

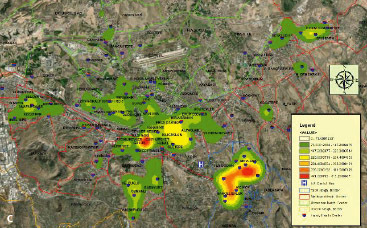
Kernel Density analyses for periodontal diseases, gingivitis, and periodontitis were conducted with a bandwidth of 1000 meters and a cell size of 83x83 meters.

In Fig 4a, KD analysis was applied to the entire 47,757 patients. Density analyses in Figs 4b and 4c were performed only for gingivitis (24,834 patients) and periodontitis (17,651 patients) without including patients from the “Other” group.

The dense neighbourhoods were evaluated at the provincial level. Patient attendance at the Faculty of Dentistry are statistically significantly higher from Mevlana Neighbourhood in Talas district and Cumhuriyet Neighborhood in Melikgazi district (Fig 5a).

**Fig 6:**
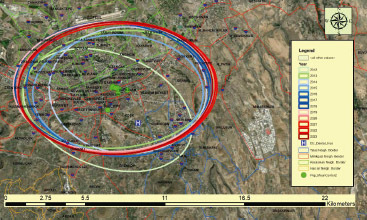
In the third stage of the analysis, standard deviation ellipses were created based on the patient population for 2012–2023. These ellipses assist us in understanding the spatial changes in patient distribution over the years. For easy comprehension, 2012–2013 are represented in green and its shades, 2014–2018 in blue and its shades, and 2019–2023 in red and its shades.

In the third stage of the analysis, standard deviation ellipses were created based on the patient population for 2012–2023. When examining the standard deviation ellipses over the years, it is observed that the spatial distributions of patients attending the Faculty of Dentistry have shown a similar pattern in the last five years (Fig 6).

## DISCUSSION

Periodontal diseases constitute important issues for public health. The silent and chronic nature of periodontal diseases, progressing for years with either asymptomatic or minimal symptoms, may lead patients to not require treatment for many years.^38^ However, in a systematic review conducted by Buset et al5 in 2016, it was shown that the functional, psychosocial, and pain/discomfort aspects of these diseases statistically significantly affect individuals’ quality of life. In recent years, advancements in GIS and KD analyses have increased access to spatial data, providing opportunities to utilise spatiotemporal data in epidemiology.^19^ Innovative methods like these aimed at improving public health facilitate ease in the diagnosis, treatment, and elimination of diseases.

This study is a pilot implementation proposing a new and useful method (using GIS and KD analyses) for epidemiological research. This study, where we generated location-based estimates for local-level periodontal disease prevalence, will be useful for future applications at the national level. Additionally, more detailed surveillance studies can be conducted that include environmental factors and systemic diseases.

Furthermore, to our knowledge, this is the first study to evaluate the geographic distribution of individuals with periodontal disease in conjunction with density analyses.

In the analysis of demographic data, there is a predominance of female patients. This situation may be associated with the region’s male-dominated workforce, women primarily being homemakers, and their ability to find time for hospital visits. Our study shows that the prevalence of periodontitis is higher in male patients than in female patients. The difference in daily routines, such as higher smoking rates among men, shorter toothbrushing durations, or a lack of awareness about oral health, may contribute to this disparity. Similar results have been found in studies conducted in other countries.^11,37^ In recent studies, it has been shown that men have a weaker immune system, which could be another factor.^24,25^

There was a statistically significant decrease in the number of patients applying between 2019 and 2021, followed by a sudden increase in 2022. This situation may be attributed to the global impact of the Covid-19 pandemic caused by the Sars-Cov-2 virus.

Additionally, studies have shown that periodontal pathogens may exacerbate the systemic effects of COVID-19, and individuals who regularly brush their teeth and use mouthwash are less likely to contract COVID-19.^1,34^ The consistent decrease in periodontitis prevalence since 2019 may also be associated with patients placing greater importance on oral health to reduce the effectiveness of the virus. Patients may have become more conscious about oral hygiene and health compared to previous years.

The patient population visiting the Faculty of Dentistry care is predominantly adults aged 36 to 55. In this age group, there is a statistically significant increase in the prevalence of periodontitis. This situation can be explained by the negative impact of chronic diseases that typically occur at older ages (e.g., diabetes, osteoporosis), smoking habits, or the general effect of aging on periodontal tissues.^15,35^

Not every case of gingivitis progresses to periodontitis, but every instance of periodontitis usually develops from gingivitis. Therefore, treating gingivitis is crucial to prevent the progression of the disease to periodontitis.^28^ When examining the distribution of the patient population over the years, the graphs of gingivitis and periodontitis show great similarity (Fig 2). Additionally, this similarity has been observed in the Kernel density maps obtained through GIS (Figs 5b and 5c). The KD analysis shows the spatial distribution of patients admitted to Erciyes University Dentistry Faculty over a 12-year period on a map of Kayseri. These maps could help select a pilot health facility in areas with a high population density of gingivitis and periodontitis for conducting routine dental examinations and providing necessary oral hygiene education. For example, Fig 5a shows a notable number of patient admissions to the Faculty of Dentistry from Mevlana Neighbourhood in Talas District and Cumhuriyet Neighborhood in Melikgazi District. Family dentistry units could be established in these neighbourhoods at Anayurt Family Health Center and Sahabiye Family Health Center. Through this (due to the reversible nature of gingivitis), dental hygiene education provided in family health centers could eliminate the disease, prevent its progression, and reduce the prevalence of periodontal disease in the community. Furthermore, patients with periodontitis who lack awareness, or have never received treatment, could be identified and referred to more comprehensive healthcare centers (e.g., dental faculties) for treatment. This approach could help prevent further gum recession, bone loss, and tooth loss.

In Turkey’s healthcare system, family health centers are local healthcare facilities where family medicine services are provided by one or more family physicians (specialists or general practitioners) with the assistance of healthcare personnel such as midwives, nurses, health officers, and medical secretaries. However, they do not contain any dental units or dentists. Thus, the establishment of dental units in family health centers will create new employment opportunities for dental hygienists and general dentists.

GIS-supported density analyses offer detailed regional insights into patient distribution based on treatment center locations. When patient density maps are examined, it is evident that there is a higher number of applications from areas closer to the Faculty of Dentistry. With increasing distance from the Faculty of Dentistry, the number of attendees also decreases. Socioeconomic status, occupational commitments (such as livestock farming and agriculture in rural areas), and childcare are reasons that may prevent patients from seeking treatment at a more distant center. It is understood that the distance to the hospital influences patients’ preferences for treatment options in this situation. Similar results were found in a study conducted by Jeong et al^19^ in Korea regarding patient distribution with CBS, and Eke et al^12^ in the USA, both of whom used geospatial analysis. Performing these analyses in more peripheral districts in future studies can help identify patients’ needs for treatment and facilitate the establishment of dental healthcare facilities tailored to those needs.

When examining standard deviation ellipses over the years (Fig 6), it can be inferred that many patients who attended were from similar spatial regions (same neighbourhood or building). Additionally, as population density centers can be determined with standard deviation ellipses, the ideal location for the Faculty of Dentistry in terms of proximity can be identified. In this manner, decisions about the areas of future dental healthcare facilities can also be determined.

However, our study has some limitations. One limitation is that our analyses did not include clinical attachment loss and pocket depths, which are essential for epidemiological studies of periodontal diseases. Between 2012 and July 2023, indications for patients attending the Oral Diagnosis and Radiology Clinic were determined solely based on radiographic patient records and clinical examinations without conducting a comprehensive periodontal examination. During the clinical examination, the periodontal assessment is conducted by measuring bleeding on probing and pocket depth using a Williams-Hu Friedy probe; however, these findings are not recorded. These examinations are performed to establish preliminary diagnoses, such as gingivitis/periodontitis/gingival recession/gingival enlargement, and are only used to refer patients to the periodontology clinic. Once patients begin treatment in the periodontology clinic, all clinical parameters are examined in detail and recorded. Before 2017, it was challenging to adapt diagnoses according to the new classification. Therefore, periodontitis was generally categorised as any form of periodontitis (without gradations of mild, moderate, or severe). Additionally, multiple physicians have conducted diagnoses and assessments. In future studies, it would be more appropriate to have complete periodontal diagnostic records and conduct standardised examinations by a single physician.

The prevalence of gingivitis has been observed to be lower than expected. This could be due to gingivitis patients excluded from the study, such as those with gingival abscess, gingival recession, gingival enlargement, etc. Additionally, the constrictive effects of smoking on gingival inflammation in smokers may mask this, which could be another factor.

Furthermore, it is not known whether the patients still reside at the same address. Due to patient confidentiality, obtaining address information on a household basis is impossible. As a result, household locations on the maps could only be obtained using city-district and street-building data.

In future studies, it may be possible to provide more extensive data related to patients, such as socioeconomic status, systemic health conditions (HbA1c levels), oral hygiene habits (daily toothbrushing frequency, flossing, etc.), and smoking status. Additionally, this study was conducted using data from a single center (Erciyes University Faculty of Dentistry, Kayseri, Turkey). It is possible to conduct future multicenter or even nationwide studies using GIS.

## CONCLUSION

Geographic Information Systems (GIS) offer numerous advantages and can be utilised in the field of dentistry:

- Hospital site selection and practical location choice: Dentists or dental clinics can choose an appropriate location by analysing the potential patient population.
- GIS can assist in assessing patient demands and competition situations in specific geographical regions. Additionally, it can help determine the optimal locations for dental clinics.
- Patient population analysis: GIS can be used to analyse the patient population in the geographic region served by a dentist or clinic. This can help clinics gain more information about their target audience and customize their services accordingly.
- Epidemiological studies: GIS can be used to examine the prevalence and distribution of dental diseases. For example, mapping the rates of periodontal disease, caries, etc, in a geographic region can assist in the formulation of region-specific health policies.
- Institutional collaboration and emergency preparedness: GIS can facilitate collaboration among dental clinics, hospitals, and other healthcare institutions, as well as prepare for emergency management. Geographic data can contribute to emergency planning, especially for natural disasters or pandemics.
- Monitoring treatment outcomes: GIS can monitor patients’ treatment outcomes and analyse treatment data geographically. This can help in understanding dental health trends in a specific region.
- Community health education and awareness: GIS can be used to identify and geographically focus on the target audience for community health education and awareness campaigns. For instance, geographic analysis can be conducted to make oral health awareness activities more effective in specific regions.
- Research and academic studies: Dental researchers can conduct studies on dental health using geographic data. This can facilitate research on causes, spread, and impacts of disease.
- GIS in dentistry can contribute to better hospital management, improvements in public health, and the development of more effective treatment strategies. Therefore, geographic data can be a significant tool in shaping government health policies and dental practices.
